# Oral health-related quality of life in Sjögren disease and non-Sjögren *sicca*: a cross-sectional study

**DOI:** 10.1186/s41687-026-01013-4

**Published:** 2026-02-14

**Authors:** Laiz Fernandes Mendes Nunes, Lucas Tadeu Ferreira Gomes, Fernanda Aragão Felix, José Alcides Almeida de Arruda, Clarice Klem de Castro Pinheiro, Lucas Guimarães Abreu, Benjamin P. J. Fournier, Leandro Augusto Tanure, Débora Cerqueira Calderaro, Maurício Augusto Aquino de Castro, Tarcília Aparecida Silva, Sílvia Ferreira de Sousa

**Affiliations:** 1 Department of Oral Surgery, Pathology, and Clinical Dentistry, School of Dentistry, Universidade Federal de Minas Gerais, Av. Pres. Antônio Carlos, room 3204, 6627, Belo Horizonte, Minas Gerais Brazil; 2 Department of Oral Diagnosis and Pathology, School of Dentistry, Universidade Federal do Rio de Janeiro, Rio de Janeiro, Brazil; 3 Department of Child and Adolescent Oral Health, School of Dentistry, Universidade Federal de Minas Gerais, Belo Horizonte, Minas Gerais, Brazil; 4 Université Paris Cité, UMR1313 INSERM Santé Orale, Montrouge, France; 5 Reference Center of Oral Rare Diseases O-Rares, Rothschild Hospital, Public Assistance-Paris Hospitals, Paris, France; 6 Hospital das Clínicas, Universidade Federal de Minas Gerais, Belo Horizonte, Minas Gerais Brazil; 7 Locomotor System Departament, Medical School, Universidade Federal de Minas Gerais, Belo Horizonte, Brazil

**Keywords:** Keratoconjunctivitis sicca, Oral health, Quality of life, Sjögren disease, Sjögren’s syndrome

## Abstract

**Background:**

Sjögren disease (SjD) and non-Sjögren *sicca* (nSS) individuals exhibit symptoms of oral dryness. However, studies investigating factors influencing oral health-related quality of life (OHRQoL) in these groups remain scarce.

**Purpose:**

To evaluate OHRQoL in SjD and nSS patients and identify factors influencing its perception.

**Methods:**

A cross-sectional study was conducted. The short version of the Oral Health Impact Profile-14 (OHIP-14) questionnaire was administered. Bivariate analyses were performed to examine associations between independent variables and OHRQoL. Statistical significance was set at *p* < 0.05.

**Results:**

A total of 116 individuals were evaluated, including 79 with SjD and 37 with nSS. Hyposalivation was more frequent in SjD (55.7%) than in nSS (27.0%). The median overall OHIP-14 score was higher in nSS (30.0) than in SjD (22.0) (*p* = 0.027), indicating poorer OHRQoL among nSS individuals. In SjD, hyposalivation significantly worsened OHIP-14 across five domains, whereas this association was absent in nSS. A negative correlation between unstimulated salivary flow rate and clinical oral dryness score, and a positive correlation between decayed, missing, and filled teeth index and OHIP-14, were observed only in nSS.

**Conclusion:**

OHRQoL impairment was greater in nSS, but salivary dysfunction exerted stronger effects in SjD, underscoring distinct determinants in *sicca* conditions.

## Introduction

Sjögren disease (SjD) is a chronic systemic autoimmune disease characterized by progressive focal lymphocytic infiltration of the exocrine glands, leading to impaired salivary and lacrimal gland function and resulting in persistent oral and ocular dryness [1, 2]. The updated term *Sjögren disease* replaces *Sjögren’s syndrome* to more accurately reflect its defined pathogenesis and systemic nature, as endorsed by the 2023 International Rome Consensus [3] and the recent British Society for Rheumatology guideline [4]. Middle-aged and older adult women are the most frequently affected, with a female-to-male ratio of approximately 9:1. The estimated prevalence in the general population is about 0.5% [5, 6].

SjD is characterized by fatigue, musculoskeletal pain, systemic manifestations, and the development of lymphoma in approximately 2–5% of affected individuals [7, 8]. The classification criteria for SjD include a minor salivary gland biopsy demonstrating focal lymphocytic sialadenitis with a focus score ≥1, the serological presence of anti-SSA/Ro autoantibodies, symptoms of oral and ocular *sicca*, and objective evidence of ocular involvement (Schirmer test ≤5 mm/5 minutes and/or Ocular Staining Score [OSS] ≥5) or salivary gland hypofunction (unstimulated salivary flow rate ≤0.1 mL/minute) [9]. The distinct group termed non-Sjögren *sicca* (nSS) comprises individuals with *sicca* symptoms and reduced salivary and/or lacrimal gland function not attributable to autoimmune disease, occurring in the absence of clinical, histopathological, or serological features of SjD [10].

Saliva lubricates and protects both the soft and hard tissues of the oral cavity, playing a crucial role in promoting and maintaining oral health through its antibacterial and antifungal activities, as well as its buffering capacity that supports tooth remineralization [11]. It also contributes to essential oral functions, including speech, taste, digestion, and bolus formation. Alterations in saliva quality or quantity can lead to chronic oral discomfort and increase the risk of oral diseases [11, 12]. Accordingly, individuals with SjD frequently experience dental caries, non-specific mucosal ulcerations, fungal infections, and difficulties with speaking, swallowing, chewing, and wearing prostheses [13–15]. Among the most characteristic manifestations of glandular dysfunction in SjD are xerostomia and xerophthalmia [16]. The former refers to the subjective sensation of oral dryness, which may or may not correspond to objectively reduced salivary flow [17]. The latter denotes the subjective perception of ocular dryness associated with lacrimal gland hypofunction [18]. These symptoms compromise fundamental oral and ocular functions, leading to impairments in nutrition, communication, and visual comfort [16, 19].

Given that oral health is a key determinant of overall health [20], alterations secondary to *sicca* conditions can profoundly interfere with daily functioning and psychosocial well-being [21]. Oral health-related quality of life (OHRQoL) captures not only functional limitations, but also the psychological and social burden associated with chronic oral symptoms [22]. In individuals with SjD, growing evidence consistently demonstrates that xerostomia and hyposalivation are major drivers of impaired OHRQoL, with higher OHIP-14 scores correlating with reduced unstimulated salivary flow, severity of subjective dryness, and oral inflammatory burden [23–25]. Beyond salivary dysfunction, sensory disturbances such as dysgeusia, burning mouth sensations, halitosis, and difficulties in speaking, eating, and swallowing have been shown to significantly exacerbate perceived oral health impact, reinforcing the multidimensional nature of OHRQoL impairment in SjD [24]. Recent comparative evidence further suggests that individuals with nSS may experience an equal or even greater perceived deterioration in OHRQoL than those with SjD, despite less pronounced objective salivary hypofunction, underscoring the complex interplay between symptom perception, psychosocial factors, and disease attribution [26]. However, while several studies have focused on SjD, data addressing OHRQoL in nSS remain scarce and fragmented, and direct comparative analyses between these conditions are limited [15, 23, 27, 28]. Such comparisons are essential to elucidate how hyposalivation, symptom burden, and subjective illness experience differentially shape functional, emotional, and social domains of OHRQoL. Therefore, the aim of the present study was to evaluate OHRQoL in individuals with SjD and nSS and to identify factors influencing its perception.

## Methods

### Study design, period of recruitment, setting, and ethical issues

A cross-sectional study was conducted between August 2022 and December 2023. Individuals reporting xerostomia and referred to the Dentistry Clinic of the School of Dentistry, Universidade Federal de Minas Gerais (UFMG), either by the Rheumatology Outpatient Clinic of Hospital das Clínicas, UFMG, or through the Municipal Regulation System of Belo Horizonte, Brazil, were recruited. The study adhered to the Strengthening the Reporting of Observational Studies in Epidemiology (STROBE) statement [29]. Ethics approval was obtained from the Research Ethics Committee of UFMG (No. 5.676.833). Informed consent was secured from all participants, and patient anonymity was preserved in accordance with the Declaration of Helsinki.

### Patients and eligibility criteria

The study was based on a convenience sample of both sexes who presented with symptoms of xerostomia or had a previous diagnosis of SjD. Patients were classified according to the 2016 ACR/EULAR criteria [9], as endorsed by the British Society for Rheumatology guideline [4]. Individuals reporting xerostomia and/or hyposalivation who did not meet the 2016 ACR/EULAR classification criteria were assigned to the nSS group. Exclusion criteria included pregnancy, prior head and neck radiotherapy, a diagnosis of HIV infection, hepatitis C, amyloidosis, sarcoidosis, graft-versus-host disease, or IgG4-related disease.

### Data collection

Data on sociodemographic characteristics, medical history, medication use, and complaints of xerophthalmia and xerostomia, as well as histopathological findings of minor salivary glands and serological parameters (i.e., anti-SSA/Ro, anti-SSB/La, rheumatoid factor, and antinuclear antibody [ANA]), were obtained through clinical interviews or extracted from medical records. Results of the OSS and Schirmer I tests were also recorded. Assessments of the oral cavity, unstimulated salivary flow rate (USFR), and OHRQoL were also performed.

### Instruments

#### USFR

USFR, a validated and widely used method for objectively assessing resting salivary gland function, was measured under standardized conditions. Participants were instructed to refrain from drinking, eating, or smoking for at least one hour before saliva collection. During the procedure, individuals were seated comfortably and asked to expectorate all saliva into a sterile graduated plastic tube over a continuous 10-minute period, avoiding swallowing or sucking. All measurements were performed following a standardized protocol by two calibrated dentists (L.T.F.G. and F.A.F.). The total salivary volume was then measured, and hyposalivation was operationally defined as a USFR of ≤0.1 mL/min, in line with previous literature [9, 30].

#### Clinical oral dryness score (CODS)

The objective assessment of xerostomia was performed using the CODS, a validated clinical instrument that quantifies the severity of oral dryness based on observable intraoral signs indicative of salivary hypofunction [31]. Clinical examinations were performed under standardized conditions by the same two calibrated dentists (L.T.F.G. and F.A.F.), following routine clinical assessment procedures and under the supervision of two senior investigators (T.A.S. and S.F.S.). An intraoral examination was conducted for each participant using a standard dental mirror. The CODS comprises a 10-point scale, with each point corresponding to a specific feature of oral dryness, as follows: mirror sticking to the buccal mucosa, mirror sticking to the tongue, depapillated tongue, fissured tongue, glassy appearance of the oral mucosa, absence of saliva on the floor of the mouth, foamy saliva, alteration of gingival architecture, non-carious cervical lesion, and presence of debris on the palate. Each positive response was assigned a value of 1, yielding a maximum possible score of 10. Higher scores indicated greater severity of oral dryness.

#### Decayed, missing, and filled teeth index (DMFT)

Each participant underwent a comprehensive dental examination performed by two experienced dentists (L.T.F.G. and F.A.F.), who applied the DMFT index. Third molars were included in the assessment [32].

#### Oral health impact profile-14 (OHIP-14) questionnaire

OHRQoL was assessed using the OHIP-14 questionnaire [33], previously validated for use in the Brazilian population [34]. The OHIP-14 comprises 14 items distributed across seven domains: functional limitation, physical pain, psychological discomfort, physical disability, psychological disability, social disability, and handicap. Each item includes five response options: 0 = never, 1 = hardly ever, 2 = occasionally, 3 = fairly often, and 4 = very often. The total OHIP-14 score ranges from 0 to 56, with higher scores indicating poorer OHRQoL. Scores for each domain were also analyzed. Participants were asked to report how frequently they had experienced any of the problems assessed by the 14-item OHIP during the previous six months. Responses were recorded using a paper-based questionnaire, following the original scale, by two calibrated dentists (L.T.F.G. and F.A.F.) who simultaneously documented the participants’ answers. The OHIP-14 was administered after completion of the clinical oral examination and salivary flow measurements.

### Statistical analyses

The Statistical Package for the Social Sciences (SPSS) software (IBM SPSS Statistics for Windows, version 25.0; Armonk, NY: IBM Corp.) was used for statistical analyses. Descriptive and bivariate analyses were performed. The normality of quantitative variables was verified using the Shapiro–Wilk test. Bivariate analyses (Mann-Whitney test and Student’s *t*-test) were applied to compare independent variables with the dependent variable (total and domain OHIP-14 scores). Statistical significance was set at *p* < 0.05. A *post hoc* power analysis was conducted in Python using the observed Mann–Whitney test statistics, group sample sizes, and a two-sided α level of 0.05.

## Results

### Demographic and clinical characteristics of participants

The clinicodemographic characteristics of the two groups are shown in Table 1. A total of 116 individuals were included in this study; among them, 79 (68%) had SjD and 37 (32%) had nSS. In the SjD group, 74 (93.7%) participants were women, whereas in the nSS group, 32 (86.5%) participants were women. The mean age of individuals with SjD was 53.2 (±14.7) years, while that of those with nSS was 56.5 (±12.3) years. Among individuals with SjD, 15 (19%) had another autoimmune disease (eight with rheumatoid arthritis and seven with systemic lupus erythematosus), whereas 64 (81%) had primary SjD (pSS).

**Table 1:** Comparison of sociodemographic and clinical characteristics between individuals with non-Sjögren *sicca* (nSS) (*n* = 37) and Sjögren disease (SjD) (*n* = 79)

Variable	nSS (n, %)	SjD (n, %)	*p***-value**
**Sex**			
Male	5 (13.5)	5 (6.3)	0.286*
Female	32 (86.5)	74 (93.7)	
**Age (years, mean ± SD)**	56.5 ± 12.3	53.2 ± 14.7	0.247***
**Smoking**			
Yes	5 (15.6)	4 (5.9)	0.140*
No	27 (84.4)	64 (94.1)	
**Alcohol consumption**			
Yes	4 (11.1)	4 (5.6)	0.319*
No	31 (86.1)	61 (84.7)	
Ex-drinker	1 (2.8)	7 (9.7)	
**Xerostomia**			
Yes	33 (89.2)	66 (85.7)	0.771*
No	4 (10.8)	11 (14.3)	
**USFR**			
USFR ≤0.1 mL/minute	10 (27.0)	44 (55.7)	0.005**
USFR > 0.1 mL/minute	27 (73.0)	35 (44.3)	
**Parotitis**			
Yes	9 (27.3)	35 (46.7)	0.088**
No	24 (72.7)	40 (53.3)	
**Other gland swelling**			
Yes	6 (18.2)	27 (36.5)	0.071**
No	27 (81.8)	47 (63.5)	

Note: ^*^Fisher’s exact test; ^**^Pearson’s chi-square test; ^***^Student’s *t* test; SD, standard deviation; USFR, unstimulated salivary flow rate

Regarding smoking and alcohol consumption, four (5.9%) individuals with SjD were smokers and four (5.6%) reported alcohol use. Among those with nSS, five (15.2%) were smokers and four (11.1%) reported alcohol use.

Individuals in both groups reported a pronounced subjective sensation of oral dryness (85.7% in the SjD group and 89.2% in the nSS group). Using the predefined cutoff for hyposalivation (USFR ≤0.1 mL/min), a significantly higher proportion of individuals with SjD (*n* = 44; 55.7%) exhibited hyposalivation compared with those with nSS (*n* = 10; 27.0%) (*p* = 0.005) (Table 1). In contrast, similar CODS scores were observed between groups. Objective assessments also revealed comparable DMFT values. Also, 35 (46.7%) individuals with SjD and nine (27.3%) with nSS reported previous parotitis (*p* > 0.05 for all) (Table 2).

**Table 2:** Comparison of duration of *sicca* symptoms and oral characteristics between individuals with non-Sjögren *sicca* (nSS) (*n* = 37) and Sjögren disease (SjD) (*n* = 79)

Variable	nSS (mean ± SD)	SjD (mean ± SD)	*p-***value**
***Sicca*** symptoms duration (years)	4.57 (3.34)	6.90 (4.79)	0.091*
CODS	4.32 (1.76)	4.38 (1.58)	0.862*
USFR (mL/minute)	0.23 (0.19)	0.17 (0.20)	0.112*
DMFT	21.85 (8.33)	21.91 (9.58)	0.973*

Note: ^*^Student’s *t*-test. CODS, clinical oral dryness score; DMFT, decayed, missing, and filled teeth index; SD, standard deviation; USFR, unstimulated salivary flow rate

The analysis of the relationship among USFR, CODS, and DMFT revealed no significant association between USFR and either CODS (*p* = 0.748) or DMFT (*p* = 0.486) in individuals with SjD. Similarly, no correlation between USFR and DMFT was found in the nSS group (*p* = 0.329). However, a significant negative correlation was found between USFR and CODS in individuals with nSS (*p* = 0.006; β = −1.238), indicating that lower salivary flow was associated with greater clinical evidence of oral dryness in this subgroup.

Considering that several comorbidities and commonly prescribed medications are associated with SjD and may influence salivary function, xerostomia, and patient-reported outcomes [21, 35], Table 3 summarizes the comparisons between SjD and nSS regarding metabolic disease, lymphoma, and the use of corticosteroids, hydroxychloroquine, methotrexate, antihypertensives, antidepressants, and anticonvulsants. No statistically significant differences were observed between groups (*p* > 0.05 for all).

**Table 3:** Comparison of comorbidities and medication use between individuals with non-Sjögren *sicca* (nSS) (*n* = 37) and Sjögren disease (SjD) (*n* = 79)

Variable	nSS (n, %)	SjD (n, %)	*p***-value**
**Metabolic disease**			
Yes	14 (38.9)	26 (33.3)	0.673^**^
No	22 (61.1)	52 (66.7)	
**Lymphoma**			
Yes	0 (0.0)	1 (1.3)	1.000^*^
No	36 (100)	77 (98.7)	
**Corticosteroid**			
Yes	10 (27.8)	22 (28.2)	1.000^**^
No	26 (72.2)	56 (71.8)	
**Hydroxychloroquine**			
Yes	6 (16.7)	15 (19.2)	0.801^**^
No	30 (83.3)	63 (80.8)	
**Methotrexate**			
Yes	5 (13.9)	24 (30.8)	0.066^**^
No	31 (86.1)	54 (69.2)	
**Antihypertensive**			
Yes	20 (55.6)	39 (50.6)	0.689^**^
No	16 (44.4)	38 (49.4)	
**Antidepressant**			
Yes	19 (52.8)	42 (53.8)	1.000^**^
No	17 (47.2)	36 (46.2)	
**Anticonvulsant**			
Yes	5 (13.9)	13 (16.7)	0.789^**^
No	31 (86.1)	65 (83.3)	

Note: ^*^Fisher’s exact test; ^**^Pearson’s chi-square test

### Associations of OHIP-14 domains and total score with clinicodemographic variables

The total OHIP-14 score among individuals with nSS was significantly higher (30.0) than among those with SjD (22.0) (*p* = 0.027), indicating poorer OHRQoL in the nSS group. Higher scores in nSS individuals were also observed across most of the seven OHIP-14 domains (Table 4).

**Table 4:** Comparison of oral health impact profile-14 (OHIP-14) scores between individuals with non-Sjögren *sicca* (nSS) (*n* = 37) and Sjögren disease (SjD) (*n* = 79)

Domain	nSS (median, range)	SjD (median, range)	*p***-value**
Functional limitation	3.0 (0–8)	2.0 (0–8)	0.253
Physical pain	7.0 (2–8)	5.0 (0–8)	**0.026**
Psychological discomfort	7.0 (0–8)	6.0 (0–8)	**0.047**
Physical disability	3.0 (0–8)	3.0 (0–8)	0.344
Psychological disability	5.0 (0–8)	4.0 (0–8)	**0.016**
Social disability	3.0 (0–8)	1.0 (0–8)	**0.039**
Handicap	2.0 (0–8)	1.0 (0–7)	**0.021**
Overall score	30.0 (5–56)	22.0 (0–50)	**0.027**

Note: Mann-Whitney test. Bold indicates statistical significance at *p* < 0.05

The presence of hyposalivation negatively impacted the OHIP-14 scores of individuals with SjD in five domains: functional limitation (*p* = 0.022), physical pain (*p* = 0.007), psychological discomfort (*p* = 0.001), physical disability (*p* = 0.002), and handicap (*p* = 0.027). In contrast, no significant differences related to hyposalivation were observed among individuals with nSS (Table 5).

**Table 5:** Oral health impact profile-14 (OHIP-14) scores stratified by unstimulated salivary flow rate (USFR) in individuals with non-Sjögren *sicca* (nSS) (*n* = 37) and Sjögren disease (SjD) (*n* = 79)

Domain	nSS (n = 37)			SjD (n = 79)		
	USFR ≤0.1 (n = 10) (median, range)	USFR > 0.1 (n = 27) (median, range)	*p***-value**	USFR ≤0.1 (n = 44) (median, range)	USFR > 0.1 (n = 35) (median, range)	*p***-value**
Functional limitation	2.5 (0–8)	3.0 (0–8)	0.908	2.5 (0–8)	0.0 (0–6)	**0.022**
Physical pain	8.0 (3–8)	6.0 (2–8)	0.126	6.5 (0–8)	4.0 (0–8)	**0.007**
Psychological discomfort	8.0 (1–8)	7.0 (0–8)	0.140	7.0 (0–8)	5.0 (0–8)	**0.001**
Physical disability	5.0 (0–8)	3.0 (0–8)	0.161	4.0 (0–8)	1.0 (0–8)	**0.002**
Psychological disability	5.5 (0–8)	5.0 (0–8)	0.939	4.0 (0–8)	3.0 (0–8)	0.094
Social disability	4.0 (0–8)	2.0 (0–8)	0.232	1.5 (0–8)	0.0 (0–6)	0.127
Handicap	3.5 (0–8)	1.0 (0–8)	0.062	2.0 (0–7)	0.0 (0–4)	**0.027**
Overall score	35.5 (6–56)	24.0 (5–56)	0.225	27.5 (4–50)	19.0 (0–42)	**0.001**

Note: Mann-Whitney test. Bold indicates statistical significance at *p* < 0.05. USFR, unstimulated salivary flow rate

Regarding the influence of DMFT and CODS on the overall OHIP-14 score, no significant correlations were found in individuals with SjD (DMFT, *p* = 0.099; CODS, *p* = 0.891). In contrast, the nSS group showed a positive correlation between DMFT and OHIP-14 (*p* = 0.001; β = 0.031), but no significant correlation with CODS (*p* = 0.884). Because other autoimmune diseases may influence salivary flow, additional analyses comparing patients with and without primary SjD and nSS were performed, yielding similar results. These findings suggest that SjD itself, rather than SjD-associated comorbidities, accounts for the observed outcomes (data not shown).

### *Post hoc* power analysis

A *post hoc* power analysis was performed for the OHIP-14 total score (the main outcome of the study). Based on the Mann–Whitney test (*p* = 0.027), the standardized effect size was estimated as *r* = 0.21 (small-to-moderate effect), corresponding approximately to Cohen’s *d* ≈ 0.41. Considering sample sizes of 37 individuals with nSS and 79 with SjD, and a two-sided α of 0.05, the estimated statistical power was approximately 54%, indicating a moderate ability to detect true group differences.

## Discussion

Individuals with SjD and those with nSS may exhibit similar symptoms of oral dryness; however, limited evidence exists regarding how these symptoms affect OHRQoL. This study demonstrated that individuals with nSS experienced a greater impact on OHRQoL, presenting worse outcomes compared with those with SjD. Importantly, while hyposalivation negatively influenced OHIP-14 scores among individuals with SjD, this association was not observed in those with nSS.

Oral health influences an individual’s social and emotional experience, as well as physical functioning, and constitutes an integral component of general health. OHRQoL has a multidimensional nature and was developed to assess how oral conditions affect patients’ overall well-being. One of the most widely used instruments to evaluate OHRQoL is the OHIP-14 [22]. Because hyposalivation and xerostomia can simultaneously affect the physical, functional, and psychosocial dimensions of oral health, all seven OHIP-14 domains were analyzed to capture the multidimensional impact of dryness symptoms on patients’ daily lives.

Previous studies have demonstrated that individuals with SjD exhibit a more negative perception of their OHRQoL compared with healthy controls [36, 37]. However, the literature addressing OHRQoL in patients with nSS remains barely explored [27]. Herein, individuals with SjD showed a higher frequency of hyposalivation and worse dryness scores, whereas those with nSS exhibited higher OHIP-14 scores. This finding indicates that individuals with nSS had a more negative perception of their OHRQoL than those with SjD, likely influenced by factors other than oral dryness. Similar results were reported in another study, in which individuals with nSS exhibited greater OHRQoL impairment than those with SjD, even when objective signs of glandular dysfunction were milder [28]. One possible explanation is that patients with nSS often do not receive comprehensive care or regular follow-up, as they do not meet the classification criteria for SjD and may experience psychological distress due to the lack of a definitive diagnosis. Moreover, discrepancies between subjective xerostomia and objective salivary flow have been well documented, suggesting that qualitative alterations in saliva or neural sensitization may exert a stronger influence on patient perception than secretion volume alone [28, 38]. Likewise, in polymedicated patients, xerostomia severity and OHRQoL impairment were more strongly associated with subjective discomfort and medication profile than with objective salivary flow, emphasizing the multifactorial and perceptual nature of oral dryness [39]. Interestingly, in a study conducted by the Sjögren’s International Collaborative Clinical Alliance, individuals with SjD exhibited better physical and mental component summary scores and a lower risk of depression compared with nSS individuals presenting symptomatic dryness. Among symptomatic patients, having a definitive diagnosis may help individuals better understand their condition and develop coping mechanisms to manage their symptoms [40].

Individuals presenting with dry mouth should be evaluated to determine whether their symptoms result from an objective reduction in salivary flow (hyposalivation) or from a subjective sensation (xerostomia). It is also essential to rule out other conditions (e.g., burning mouth syndrome), particularly in women over 40 years of age with equivalent demographic characteristics [41]. Additional causes, including dehydration, trauma, alterations in afferent stimuli, central nervous system disorders, chronic salivary gland inflammation, medication use, tobacco consumption, recreational drug use, systemic diseases, and prior head and neck radiotherapy, should likewise be considered [14, 42].

Xerostomia and hyposalivation are closely associated with SjD and result from functional and structural alterations in the salivary glands, leading to several subjective and clinical manifestations due to a reduction in both the quantity and quality of saliva. These manifestations include an increased risk of dental caries, infection (e.g., oral candidiasis), difficulty wearing dentures and chewing, dysphagia, dysgeusia, speech impairment, oral mucosal lesions, and salivary gland inflammation [14, 42]. Therefore, multidisciplinary care involving dentists, rheumatologists, nutritionists, physical therapists, and psychologists is highly recommended [43]. This multiprofessional approach is reinforced by the recent SjD guideline from the British Society for Rheumatology [4]. The guideline advises patients to adopt several lifestyle measures, including long-term disease monitoring, wearing glasses to reduce tear evaporation, omega-3 supplementation, avoiding dry and smoky environments, maintaining ambient humidity (e.g., reducing heating or placing saucers of water on radiators), reducing sugar intake, maintaining meticulous oral hygiene, and drinking plain water regularly [4].

Unlike individuals with SjD, for whom multidisciplinary care protocols have been established, there are no standardized guidelines for the treatment or monitoring of dry mouth in nSS individuals. However, it is important to recognize that, over time, the criteria and classification for SjD have evolved, and patients previously diagnosed with SjD may have lost this designation under the 2016 criteria. Additionally, individuals currently categorized as nSS may represent early or incomplete forms of SjD. Therefore, continuous clinical surveillance of individuals with nSS is essential, as longitudinal reassessment may lead to diagnostic reclassification. In addition, given that these patients experience poorer OHRQoL, studies proposing multidisciplinary approaches and standardized management guidelines are warranted. In this scenario, OHRQoL assessment serves as a valuable tool for capturing the subjective burden of oral dryness beyond objective measures, enabling clinicians to identify unmet needs, guide supportive care, and advocate for the appropriate allocation of health resources in this population.

There was no significant association between USFR and OHIP-14 scores among individuals with nSS in our study. It has been suggested that alterations in saliva quantity are not the main factors associated with poor OHRQoL, and that other aspects—particularly saliva quality—also deserve attention. Saliva is composed primarily of water, along with varying concentrations of electrolytes and a complex mixture of proteins, glycoproteins, enzymes, and other molecules with biological and biochemical properties essential for maintaining oral physiological homeostasis. Each salivary component performs specific functions, including lubrication, antimicrobial activity, maintenance of mucosa integrity, phonation, cleansing, and other protective roles [11, 12]. Saliva forms a thin film that covers both hard and soft tissues, providing protection and moisture to the oral, oropharyngeal, and esophageal mucosae. Its composition varies according to the type of salivary gland involved and the source of stimulation [42]. Further studies are required to elucidate how changes in salivary composition influence oral dryness and the OHRQoL of individuals with nSS.

In contrast to individuals with nSS, patients with SjD and hyposalivation exhibited significantly worse OHRQoL, as previously documented elsewhere [23, 27]. Salivary secretion is a regulated physiological process that occurs predominantly in response to stimulation. Mechanical and chemical stimuli are the most effective, with eating and chewing serving as the primary triggers of salivary flow. Between meals, salivation proceeds slowly and decreases to nearly zero during sleep. Patients with reduced salivary flow are susceptible to alterations in both soft and hard oral tissues, as well as to changes in the oral microbial load. Although some studies have demonstrated that total salivary bacterial counts are similar between patients with SjD and healthy controls [44], low salivary flow has been associated with a microbial shift involving species such as *Streptococcus mutans* and *Candida albicans* [45, 46]. This may explain, in part, the higher occurrence of dental caries and oral candidiasis in patients with SjD. Conversely, periodontal microorganisms appear to remain unaltered in SjD [42].

In the present study, although 55.7% of individuals in the SjD group exhibited hyposalivation, similar CODS and DMFT values were observed between the groups. However, a negative correlation between USFR and CODS was identified only in the nSS group. This divergence from previous reports may reflect differences in sample composition, disease severity, or methodological protocols used for saliva collection and CODS scoring [9, 28, 31]. It is reasonable to suggest that, in our study, CODS may not have perfectly paralleled USFR, as the clinical manifestations of dryness can appear later than functional alterations, particularly in early-stage or nSS cases. In contrast to our findings, earlier studies have shown that individuals with SjD exhibit significantly higher mean CODS values than those with nSS [28, 46].

The literature has demonstrated that DMFT values are higher in SjD patients than in healthy controls [47]; however, to date, no studies have compared DMFT between SjD and nSS patients. It is well established that dental caries is a multifactorial disease influenced by both salivary flow and composition, including ionic and protein constituents. When these factors are altered, they become significant risk factors for the development of dental caries [48]. In this context, systematic reviews have corroborated the general concept that a higher dental caries burden is associated with poorer OHRQoL. Haag et al. [49] reported that all studies evaluating dental caries found a negative association with health-related quality of life. Of note, individuals with dental caries had approximately 3.5-fold higher odds of poor OHRQoL than those without lesions [50]. This reinforces that a higher DMFT score, as a proxy for cumulative dental caries experience, can reasonably be expected to contribute to poorer OHRQoL. Although no direct association between USFR and DMFT was identified in either group, it is plausible that DMFT mediates or moderates the pathway linking hyposalivation to perceived oral health impact.

The limitations of this study include its cross-sectional design and the use of a convenience sample drawn from a single tertiary health service in Brazil. Although recruitment occurred over a representative period and the center receives patients from different regions, the overall sample size (particularly in the nSS group) was modest. Consequently, the study may have been underpowered to detect subtle associations, especially in analyses involving subgroup comparisons. Therefore, the findings should be interpreted with appropriate caution. Nonetheless, given the relative rarity and clinical heterogeneity of SjD and nSS, the sample reflects real-world clinical practice.

## Conclusion

In summary, individuals with nSS exhibited a more negative perception of OHRQoL compared with those with SjD. Among individuals with SjD, hyposalivation and longer duration of dryness symptoms were associated with poorer OHRQoL. These findings underscore the need for improved care and multidisciplinary management of patients with SjD, as well as continued clinical surveillance of nSS individuals. Identifying specific determinants in nSS remains essential for developing targeted interventions.
